# Improving Prescribing Practices in Primary Care: A randomised trial and economic analysis of a multicomponent intervention showed small, but important, gains

**DOI:** 10.1371/journal.pmed.0030229

**Published:** 2006-06-06

**Authors:** Chris Del Mar

## Abstract

Del Mar discusses the implications of the RaPP trial, a cluster RCT of a multi-component intervention to improve prescribing of antihypertensive and cholesterol-lowering drugs in primary care.

In a new paper in *PLoS Medicine*, Fretheim and colleagues report the results of the Rational Prescribing in Primary Care (RaPP) trial, a cluster-randomised controlled trial of a multicomponent intervention to improve prescribing of antihypertensive and cholesterol-lowering drugs in primary care [ 1]. In a second paper, the same authors present their economic evaluation of the intervention [ 2].

In the trial, the unit of randomization was the general practice, and the authors randomized 146 general practices from two geographical areas to the intervention or control. The multicomponent intervention included educational outreach visits to clinics to discuss clinical practice guidelines, an audit and feedback on current adherence to guidelines, and computerized reminders to doctors during patient consultations. The control intervention was passive dissemination of guidelines through a national medical journal.

## Trial Outcomes

The trial was meticulously executed, with large numbers and a well-designed intervention. Essentially, it was a negative trial. The intervention had no impact upon two major outcomes: (1) the proportion of patients in whom cardiovascular risk was estimated, among all those started on antihypertensive or cholesterol-lowering treatment for primary prevention, and (2) the proportion of patients with a recorded level of cholesterol (total or low-density cholesterol) or blood pressure satisfying the specified treatment goals, among all patients on the corresponding treatment for at least three months. The only positive outcome was that the intervention led to increased prescribing of thiazide diuretics as a first-line treatment, something that is already being addressed as a policy issue by the government in Norway and in a number of other countries.

Drug ads will tend to have a greater effect on doctors' prescribing than exhortations to use cheaper drugs.

So on the face of it, this is a negative trial. Doctors were no more likely to use cardiovascular risk assessment, even when provided with pop-up reminders on their computerized medical records and with software designed to provide the necessary calculations automatically.

## Implications of the Study

However, the increase in prescribing of thiazide diuretics as a first-line treatment for the hypertension that contributed to increased cardiovascular risk needs careful consideration. The increase was by 12% overall, increasing the total rate from 5% to only 17% of patients in the intervention group. At first, this must seem very disappointing. As a profession, we doctors underuse a very effective and safe drug, and despite the development of much more expensive alternatives—heavily promoted by the drug industry—thiazide is the drug of first choice. Thus, 17% seems a very modest achievement with so much effort. One might be forgiven for becoming despondent.

And yet this multicomponent intervention is perhaps the best attempt that could ever be made at increasing the quality of care for a single condition in primary care. The intervention seems to have covered all bases, including using print material and guidelines [ 3], audit and feedback [ 4], and “outreach” visits [ 5]—each of which the literature is only mildly optimistic about. Moreover, hypertension is the most common chronic condition requiring long-term prescribing in primary care. Little more could have been done without invoking draconian measures, such as legislation.

The results of this study seem to fit with the recurrent theme of doctors' professional behaviour being hard to shift [ 6]. Doctors often work by heuristics (i.e., using rules of thumb and trial and error) rather than always following established care pathways. And when it comes to caring for patients, doing more (e.g., prescribing the latest brand name antihypertensives) may just feel better than doing less (e.g., prescribing an “old fashioned” thiazide), even if doing more turns out to be more costly. If this is true, then drug industry advertising will tend to have a greater effect upon doctors' prescribing patterns than exhortations to use cheaper drugs.

Were such small gains worth the effort? The answer comes in the second paper [ 2]. An economic analysis finds that although the costs of the intervention were much more than the savings represented by the use of thiazides rather than the more expensive alternatives in the short term, one only had to wait two years for the savings to outweigh the costs. This two-year cost saving is astonishing for such a small change in prescribing.

## Conclusion

It seems that the effort of throwing everything at doctors (except, perhaps, financial incentives [ 7]) is only very modestly effective in terms of improved clinical behaviour. However, it is nevertheless cost-effective. More than that, it is cost saving. And that being the case, it is almost criminal not to implement the intervention everywhere. We must surely also explore whether the intervention would be beneficial for managing other conditions in primary care.

And as a final thought, these economic equations make one realise why drug companies spend so much money on persuading doctors to use their modern but expensive alternatives. In this case, the cost differential was as beneficial to their shareholders as the savings were to the Norwegian government.

## Abbreviation

RaPP: rational prescribing in primary care
